# Using prediction markets of market scoring rule to forecast infectious diseases: a case study in Taiwan

**DOI:** 10.1186/s12889-015-2121-7

**Published:** 2015-08-11

**Authors:** Chen-yuan Tung, Tzu-Chuan Chou, Jih-wen Lin

**Affiliations:** Graduate Institute of Development Studies, National Chengchi University, 64, Zhi-Nan Road, Sec. 2, Wenshan, Taipei, 11605 Taiwan; Center for Prediction Markets, National Chengchi University, Taipei, Taiwan; Institute of Political Science, Academia Sinica, Department of Political Science, National Chengchi University, Taipei, Taiwan

## Abstract

**Background:**

The Taiwan CDC relied on the historical average number of disease cases or rate (AVG) to depict the trend of epidemic diseases in Taiwan. By comparing the historical average data with prediction markets, we show that the latter have a better prediction capability than the former. Given the volatility of the infectious diseases in Taiwan, historical average is unlikely to be an effective prediction mechanism.

**Methods:**

We designed and built the Epidemic Prediction Markets (EPM) system based upon the trading mechanism of market scoring rule. By using this system, we aggregated dispersed information from various medical professionals to predict influenza, enterovirus, and dengue fever in Taiwan.

**Results:**

EPM was more accurate in 701 out of 1,085 prediction events than the traditional baseline of historical average and the winning ratio of EPM versus AVG was 64.6 % for the target week. For the absolute prediction error of five diseases indicators of three infectious diseases, EPM was more accurate for the target week than AVG except for dengue fever confirmed cases. The winning ratios of EPM versus AVG for the confirmed cases of severe complicated influenza case, the rate of enterovirus infection, and the rate of influenza-like illness in the target week were 69.6 %, 83.9 and 76.0 %, respectively; instead, for the prediction of the confirmed cases of dengue fever and the confirmed cases of severe complicated enterovirus infection, the winning ratios of EPM were all below 50 %.

**Conclusions:**

Except confirmed cases of dengue fever, EPM provided accurate, continuous and real-time predictions of four indicators of three infectious diseases for the target week in Taiwan and outperformed the historical average data of infectious diseases.

## Background

All public health agencies hope to forecast the outbreak and duration of infectious diseases so that preventive measures can be taken. A popular approach has been the stochastic models, in which the Bayesian models and simulations are some of the advanced methods. These methods are all based on data of infectious diseases collected in a specific time and space, and their reliability is determined by the sample size and their theoretical assumptions. Prediction market, on the other hand, is a mechanism aiming to produce correct forecasts even though the information is partial or limited.

Over the last decade or so, there are 44 studies of epidemic disease prediction models [1]. Except the papers with the prediction markets system written by Polgreen, Nelson, Neumann [2, 3], most forecasting models are based upon the past data and not real-time forecasting models. As Nsoesie, Beckman, Shashaani, Nagarai and Marathe [4] pointed out, those forecasting models could perform well as long as their assumptions are right and there are good surveillance data. Nevertheless, it is difficult to make right assumptions according to different regions around the world and acquire real-time good surveillance data in the real world.

An alternative is to understand the trend of influenza epidemics by search engine query data. Ginsberg et al. [5] and Polgreen et al. [6] used keywords in the search engines of Google and Yahoo to produce trends that are strikingly similar to the flu surveillance pattern. However, Google Flu Trends is not a prediction system. It is a surveillance system and does not provide surveillance signal for Taiwan. Nevertheless, Google Flu provides real-time surveillance information for the flu trend. Most prediction models provide neither real-time prediction nor surveillance information.

Recently, in a contest hosted by the Centers for Disease Control (CDC) of the USA to promote innovation in flu activity modeling and prediction, Shaman and his team plugged digital data from CDC’s influenza-like illness data as well as social media and internet search engine into a mathematical model and then calibrating the model to produce an accurate and reliable forecast for the timing, peak and intensity of the 2013–14 flu season. Shaman’s team tested their model against actual flu activity that had already occurred during the season. By looking at the immediate past, Shaman and his team fined-tuned the model to better predict the future. However, the USA CDC has not yet officially used any model to predict the flu activity and will continue to explore the possibilities of flu forecasting.

We spent three months to present our methodology of epidemic prediction markets (EPM) to and collaborate with the Taiwan CDC on the project drafting. In the real world of Taiwan, we were told by the CDC of Taiwan during this Taiwan CDC-sponsored project drafting period that it was very difficult to predict the trend of three particular epidemic diseases in Taiwan: influenza, dengue fever and enterovirus. In the past, voluntary medical doctors reported their cases to the CDC as early as they could, but the results were not always complete and timely. Therefore, the CDC told us clearly that they relied on the historical average number of disease cases or rate (AVG) to portray the trend of epidemic diseases in Taiwan. That was why we used the AVG as the benchmark for the comparison of prediction accuracy for the EPM.

However, we will show that historical average data serve the purpose of surveillance rather than prediction after comparing their results with the prediction markets. Given the volatility of the infectious diseases, historical average is unlikely to be an effective prediction mechanism. Taiwan CDC understood that these forecasts were not accurate, but they did not have better methods to predict the trend of epidemic diseases in the real world. To solve this problem, efforts have been made to introduce other methods to forecast the spread of infectious diseases. This Taiwan CDC-sponsored project was one of them.

Prediction markets—initiated by the University of Iowa’s Electronic Market in 1988 to predict a future event—have been proven useful for the continuous and real-time forecasting of infectious diseases [2, 3]. This paper tried to construct an epidemic prediction market (EPM) system to provide accurate, continuous and real-time prediction system for five indicators of three epidemic diseases for eight weeks ahead of the target week and seven regions around Taiwan. Since the AVG is currently the only method adopted by the CDC to depict the trend of epidemic diseases in Taiwan, we assessed the accuracy of the EPM prediction against that of the AVG.

The EPM we constructed not only provided accurate and real-time predictions as other prediction markets did for other diseases, but also improved the Iowa Influenza Market (IIM), so far the most successful prediction market of epidemics, in duration, space, the number of diseases, and the method of transaction. In addition, we were told by the Taiwan CDC that it was very difficult for Taiwan CDC to predict the trend of these three particular epidemic diseases in Taiwan: influenza, dengue fever and enterovirus. It is thus a good case to test the capability of prediction markets. Located in the subtropical climate region, Taiwan is subject to easy outbreaks of epidemics of infectious diseases. Global warming and international transportation further increases the frequency and periodicity of these diseases, hence the difficulty to predict them [7, 8].

Infectious diseases can spread across any region, but Taiwan has not been a member of the World Health Organization. There is a surveillance system of infectious diseases in Taiwan. To build up a Taiwan’s virus surveillance network, Taiwan CDC has so far commissioned 8 medical centers spread in all parts of Taiwan since 1999, and to have each set up a contract laboratory of diagnostic virology for detecting, organizing, and reporting suspected enterovirus cases as well as severe influenza ones. Besides, a separate sentinel physician-based surveillance system was also established with doctors assigned almost in each and every district and township across Taiwan, and a domestic virus strain database was assembled. However, there is no effective prediction system for these epidemic diseases. Accordingly, filling this gap by accurate data regarding Taiwan’s infectious diseases is of critical importance.

This paper will demonstrate the effectiveness of the EPM predictions of influenza, enterovirus, and dengue fever by comparing them with the historical averages released by the Taiwan government. The next two sections will describe our method and findings.

## Methods

A prediction market, operating like a futures market, is a forecasting mechanism capable of processing the dynamic aggregation of dispersed information from various participants [9, 10]. During the trading period, new information is continuously absorbed by the traders and reflected on the market prices. On the expiry date of the futures contracts, the settlement price will be determined by the outcome of the prediction event [11].

In the recent two decades, prediction markets have been proven empirically to be remarkably accurate in forecasting future events [12], such as elections [13–15], sports competitions [16, 17] and movie box offices [18–20]. Prediction markets have two major characteristics that are advantageous in prediction accuracy over traditional methods of prediction. The first is the incentive structure of reward and punishment, which induces participants to provide real and effective information and reduces market manipulation in most situations [21, 22]. The second is the continuous update of information for the predicted events, so that participants conduct trade online to provide real-time information.

**Fig. 1:**
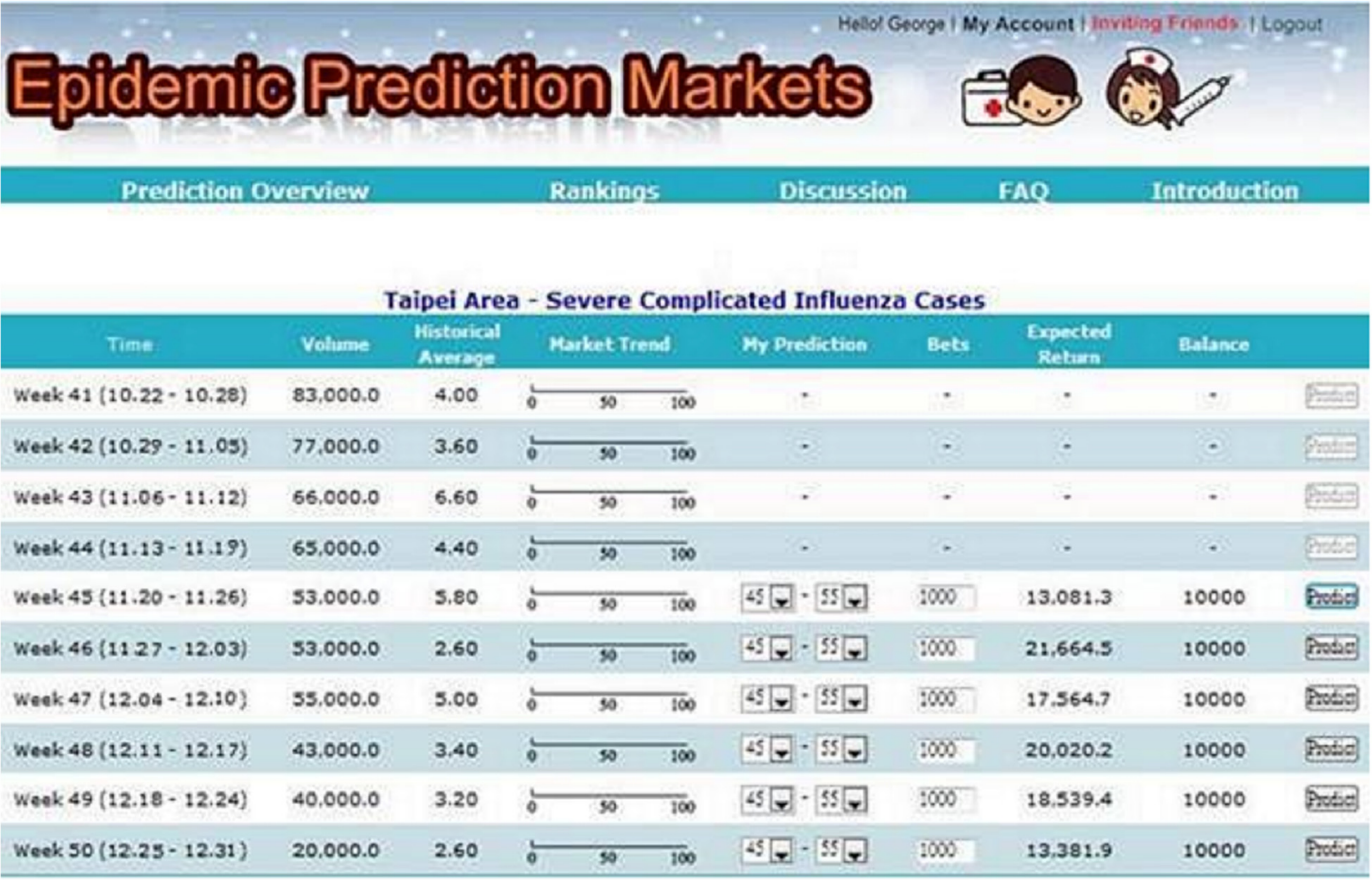
Epidemic prediction markets

We designed and built the first Epidemic Prediction Markets (EPM) system sponsored by the CDC of Taiwan (See Fig. 1). EPM invited medical professionals in Taiwan to participate. The registered members of EPM were encouraged to predict three epidemics with five indicators: (1) confirmed cases of severe complicated influenza case, (2) confirmed cases of dengue fever, (3) confirmed cases of severe complicated enterovirus infection, (4) rate of enterovirus infection, and (5) rate of influenza-like illness (ILI).

In order to improve trading liquidity and efficiency to aggregate information, EPM adopted market scoring rules (MSR) as the trading mechanism, instead of continuous double auction (CDA), which is often used by the stock or futures markets. Compared with CDA, MSR can avoid the thin market problems [23, 24]. This is very important to EPM as medical professionals are often too busy to actively participate in the trading.

According to the rules of EPM system, each participant was given 10,000 health dollar (H$) for each prediction event, and they were encouraged to predict the three epidemics with five indicators for the target week and seven weeks in advance and in seven areas of Taiwan. Health dollar is trading instrument to provide valuable information of epidemic diseases in the epidemic prediction market. Participants with accurate prediction would gain positive credits, and vice versa. Each trader will be ranked by the net balance of positive credits for her/his performance in the predictions. For every month of trading period, active participants would be rewarded with three awards of USD 33. Outstanding participants would be rewarded with awards of USD 1,000, USD 666, and USD 333, along with prize certificates from the CDC of Taiwan.

In this paper, we compared the forecast accuracy of two forecasting methods: the first is the historical average number of disease cases or rate (AVG) for the same weeks from 2005 to 2009. The second is the predictive value of the EPM. Since the AVG is currently the only method adopted by the CDC to depict the trend of epidemic diseases in Taiwan, we compare our prediction results with that of the effective alterative method (AVG) in the real world of Taiwan. In order to compare the accuracy, we calculate the prediction error values of these two methods as follows:

Prediction_error_value = Predicted_value – Actual_value

Lower prediction error value means higher prediction accuracy, and vice versa. When the prediction error value of EPM is less than that of AVG, the number of wins for EPM is to increase by one. Therefore, the winning ratio is calculated as follows:

Winning_ratio = Wins/Total_number_of_predictions

## Results

### Participation of EPM

From the 10th week (March 7–13) to the 40th week (October 3–9) of 2010, 630 medical professionals registered with this EPM. Nevertheless, only 126 members traded the prediction events of diseases indicators. The composition of these participants are: 48 nurse specialists, 23 doctors, 13 medical inspectors, four pharmacists, three nurses, two Chinese medicine practitioners and 33 other professionals. Regarding institutions, 40 participants were from public hospitals and another 40 from private hospitals; 21 from Taiwan’s Ministry of Health and its related units; 7 from clinics; 4 from local public health bureaus; the rest 14 participants from other units. In terms of areas where the participants worked, 38 participants came from Taipei area; 29 from southern area; 21 from northern area; 15 from Kaohsiung-Pingtung area in the south of Taiwan; 12 from eastern area; and 11 from central area. Tables 1 and 2 show the statistics of participants participating in prediction of five diseases indicators and seven areas

**Table 1:** The statistics of participants participating in prediction of five diseases indicators

Indicator	Participants	Predictions	Trading amount
Confirmed cases of dengue fever	84	4,395	29,503,332
Confirmed cases of severe complicated influenza case	81	3,956	33,501,580
Rate of enterovirus infection	76	3,495	31,627,923
Rate of influenza-like illness	64	2,660	23,759,804
Confirmed cases of severe complicated enterovirus infection	59	4,291	23,199,675
Total	126	18,797	141,592,314

**Table 2:** The statistics of participants participating in prediction of five diseases indicators in seven areas

Region	Participants	Predictions	Trading amount
Taipei area	64	3,511	24,875,745
Northern area	50	2,596	21,166,549
Central area	41	2,471	18,959,092
Southern area	48	2,517	18,385,997
Kaohsiung- Pingtung area	48	2,486	18,096,217
Eastern area	32	2,570	18,748,079
Nationwide area	49	2,646	21,360,636
Total	126	18,797	141,592,314

.

In terms of the statistics of participants participating in prediction of diseases indicators in different weeks in advance of the target week, there were 110 participants participated in the target week with 7,184 predictions (38 % of total predictions) and trading amount of H$56,840,809 (40 % of total trading amount). For one week in advance of the target week, 89 participated with 2,289 predictions (12 % of total predictions) and trading amount of H$15,623,775 (11 % of total trading amount). For 2 weeks in advance, 64 participated with 1,493 predictions (8 % of total predictions) and trading amount of H$10,082,893 (7 % of total trading amount). For the rest, please See Table 3.

**Table 3:** The statistics of participants participating in prediction of diseases indicators in eight weeks

	Participants	Predictions	Share of predictions	Trading amount	Share of trading amount
0 week in advance	110	7,184	38 %	56,840,809	40 %
1 week in advance	89	2,289	12 %	15,620,775	11 %
2 weeks in advance	64	1,493	8 %	10,082,893	7 %
3 weeks in advance	51	1,169	6 %	8,179,810	6 %
4 weeks in advance	44	1,226	7 %	8,730,681	6 %
5 weeks in advance	38	1,233	7 %	8,807,299	6 %
6 weeks in advance	34	1,478	8 %	11,148,133	8 %
7 weeks in advance	32	2,725	14 %	22,181,914	16 %
Total	126	18,797	100 %	141,592,314	100 %

### Winning ratios of two methods for all diseases and all areas

**Fig. 2:**
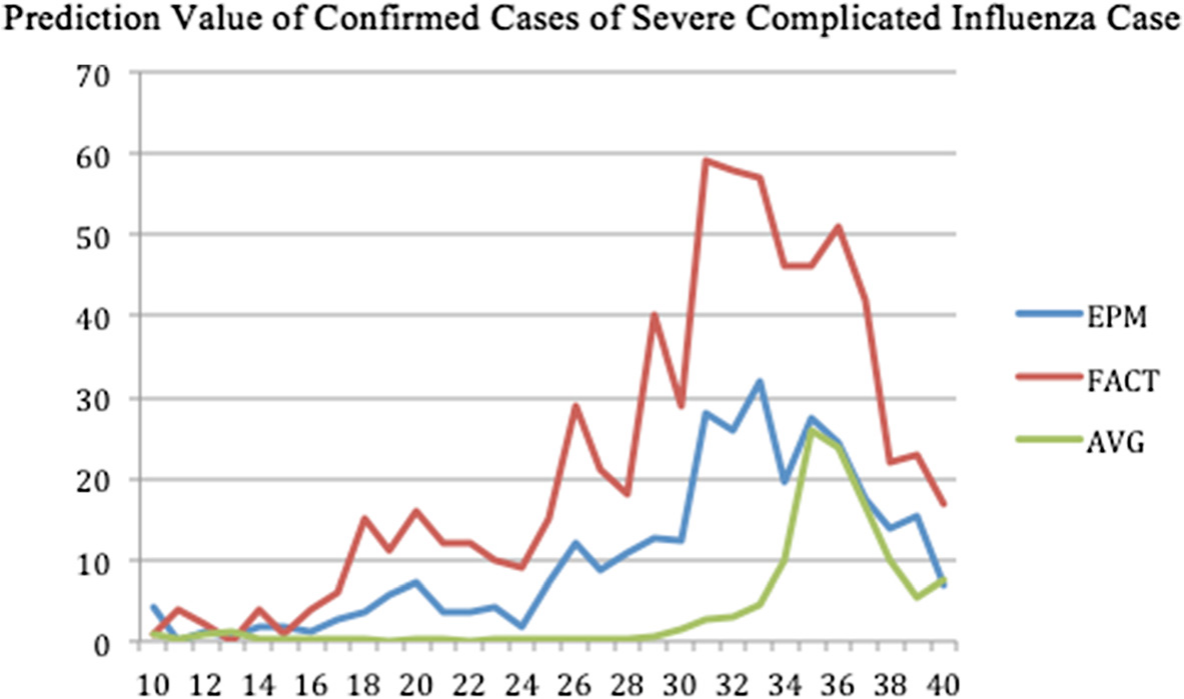
Prediction value of confirmed cases of severe complicated influenza case

**Fig. 3:**
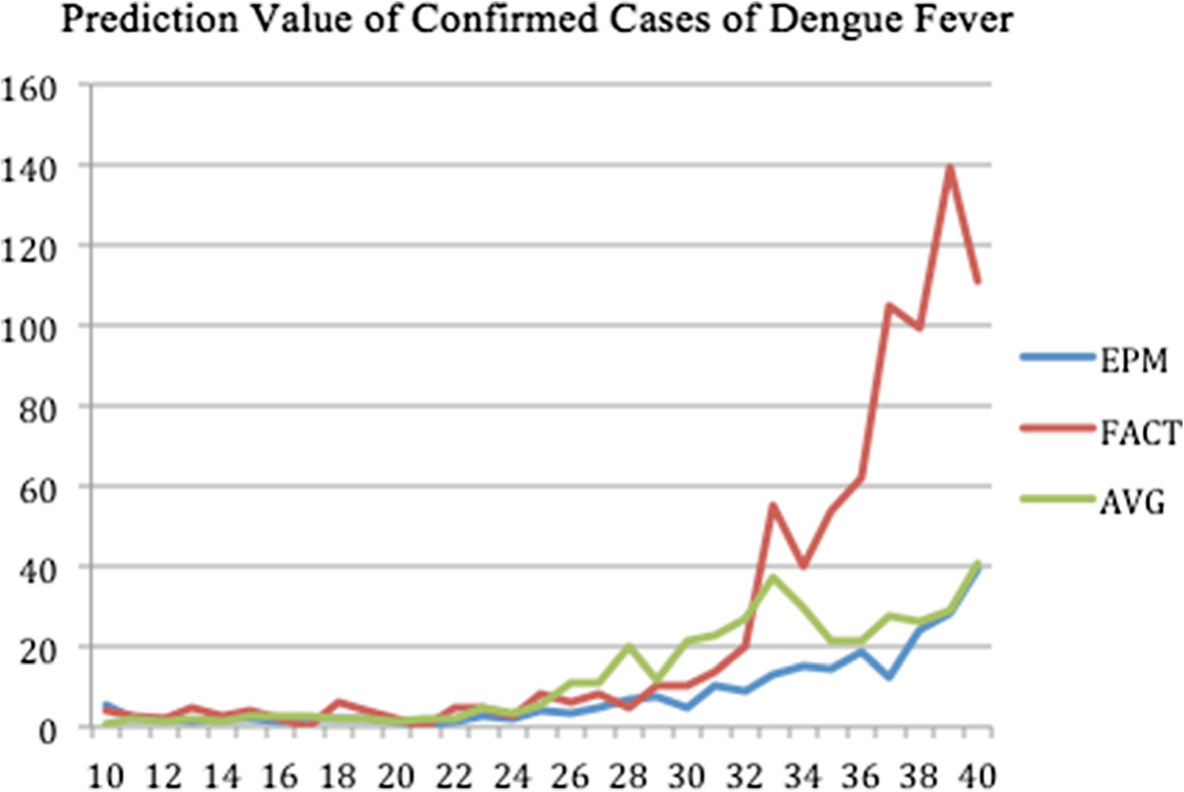
Prediction value of confirmed cases of dengue fever

**Fig. 4:**
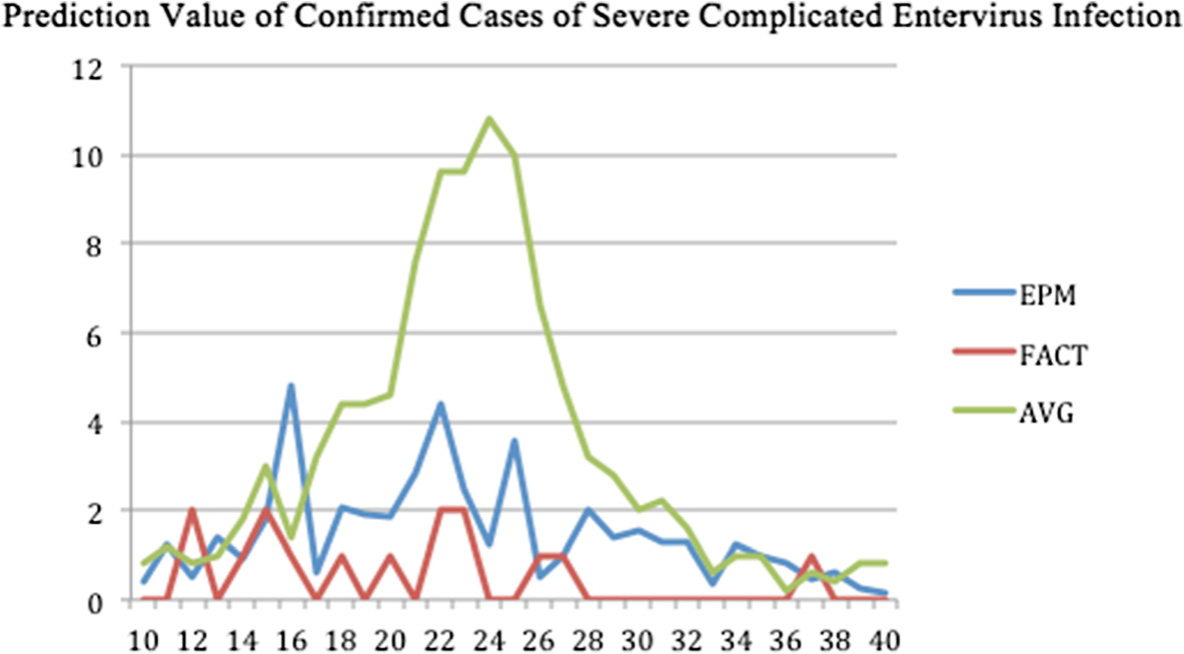
Prediction value of confirmed cases of severe complicated entervirus infection

**Fig. 5:**
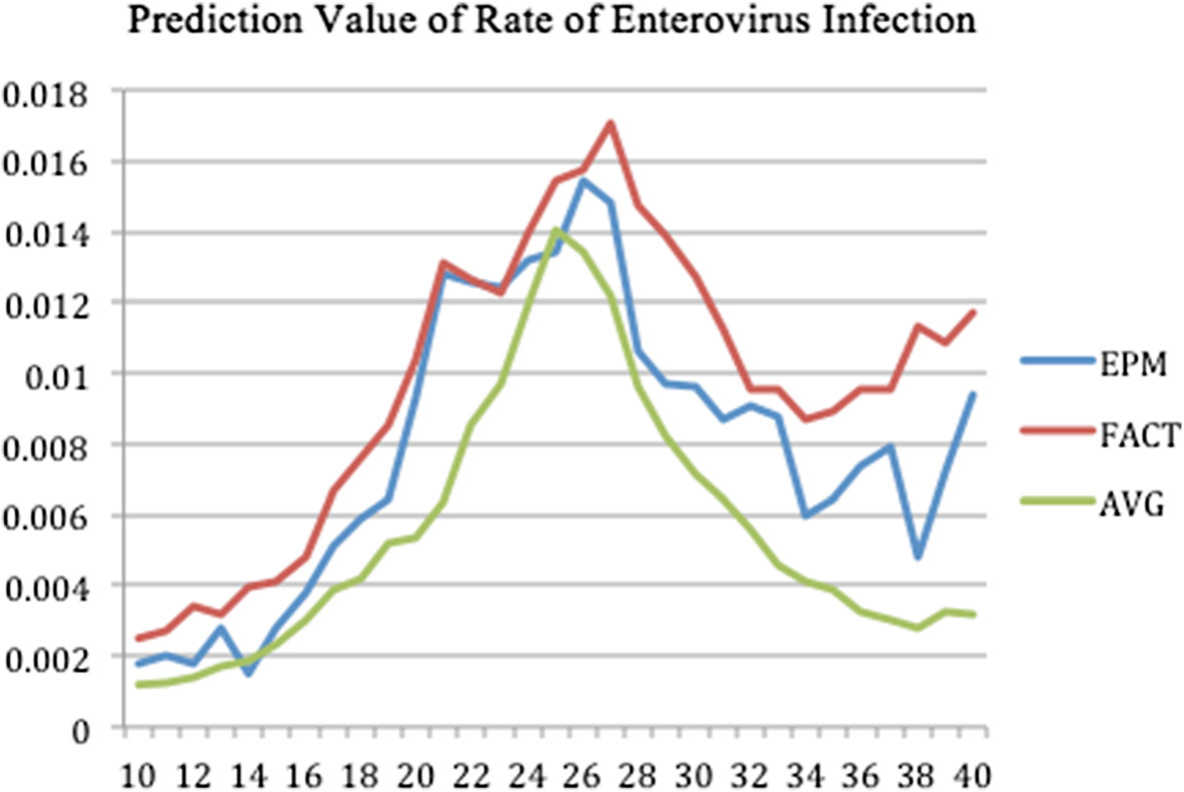
Prediction value of rate of enterovirus infection

**Fig. 6:**
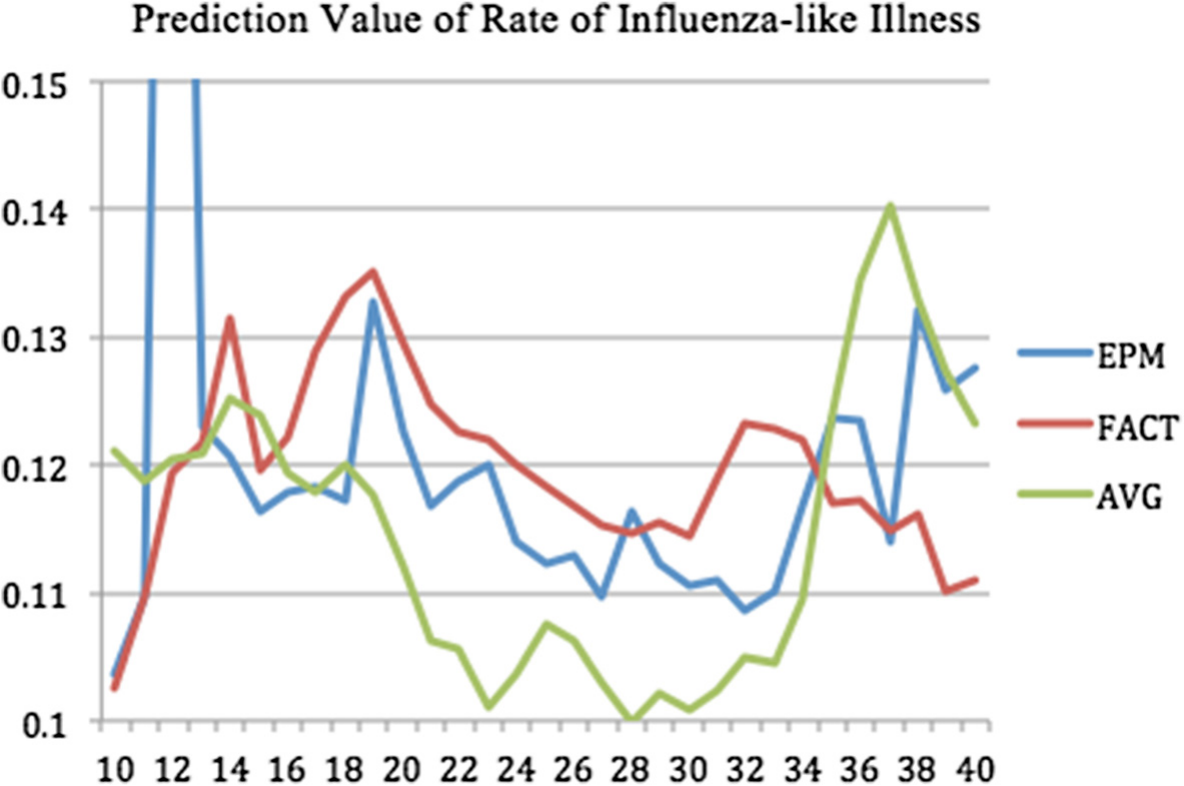
Prediction value of rate of influenza-like illness

**Fig. 7:**
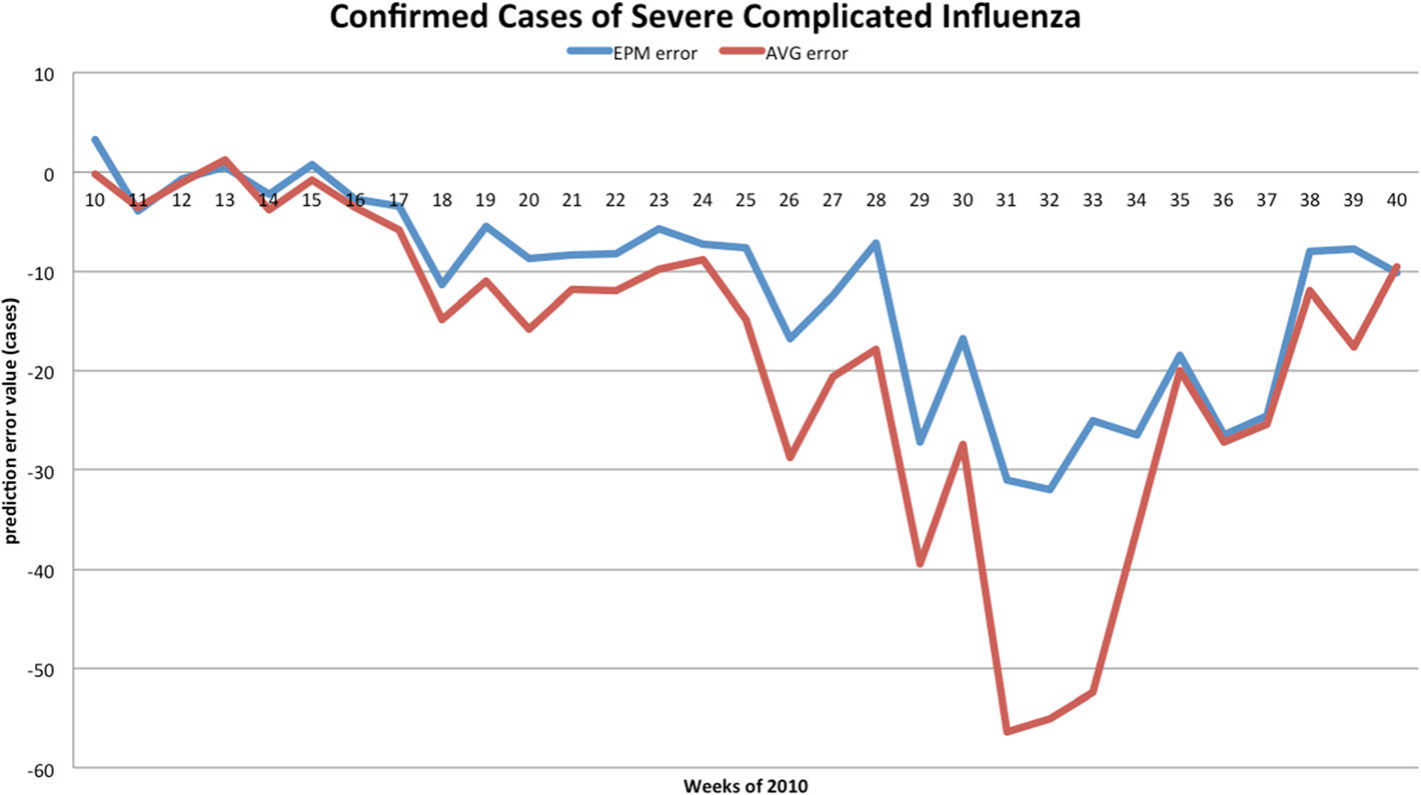
Prediction error value for confirmed cases of severe complicated influenza

**Fig. 8:**
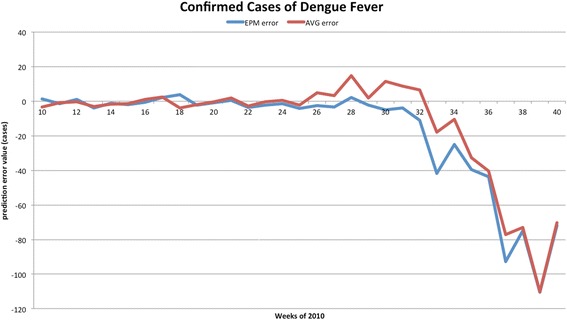
Prediction error value for confirmed cases of dengue fever

**Fig. 9:**
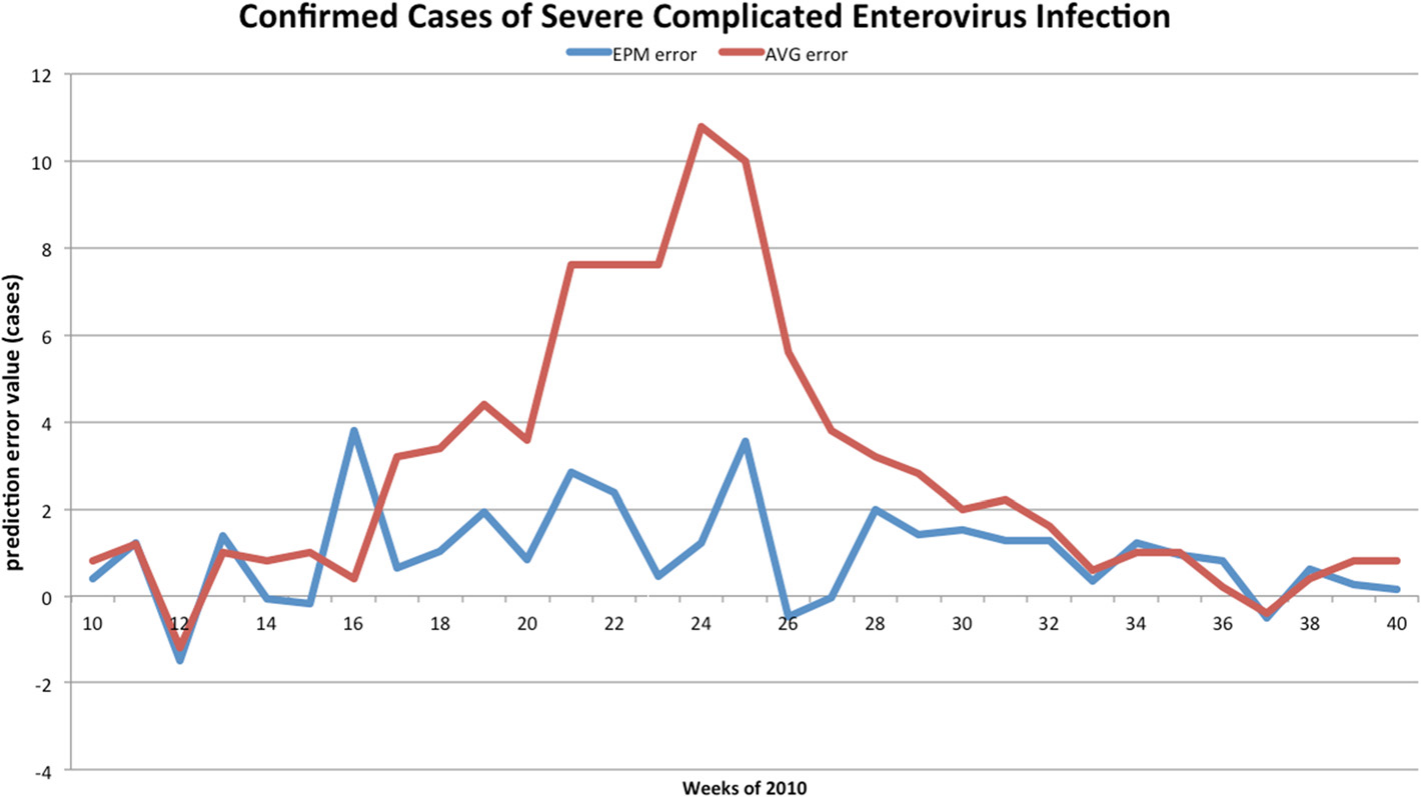
Prediction error value for confirmed cases of severe complicated enterovirus infection

**Fig. 10:**
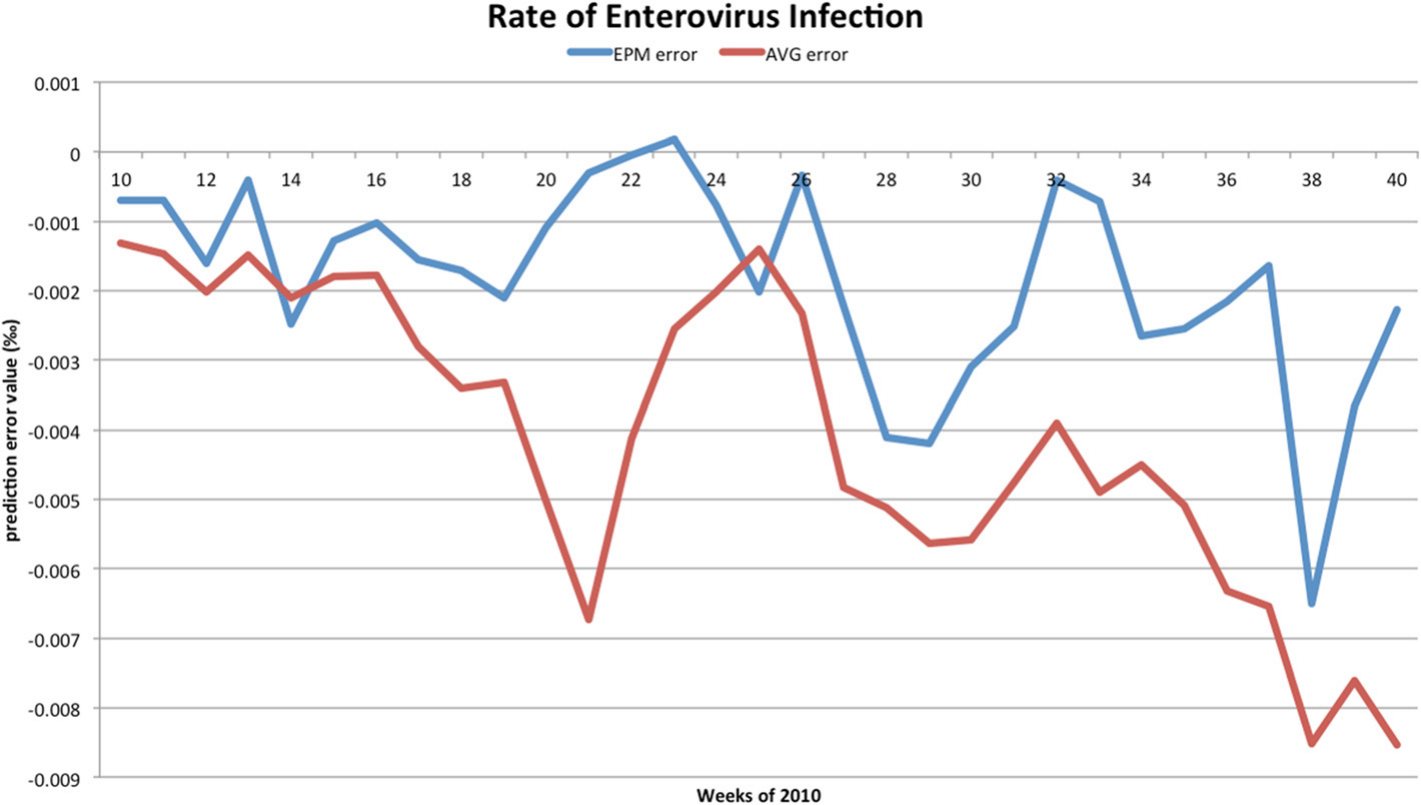
Prediction error value for rate of enterovirus infection

**Fig. 11:**
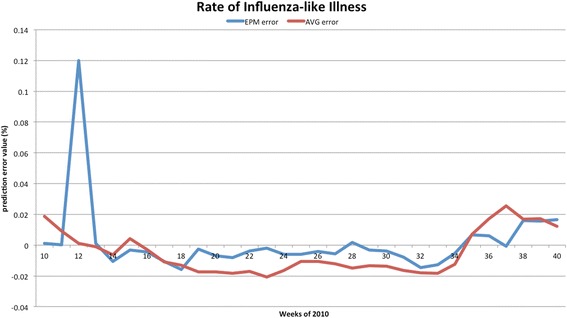
Prediction error value for rate of influenza-like illness

**Fig. 12:**
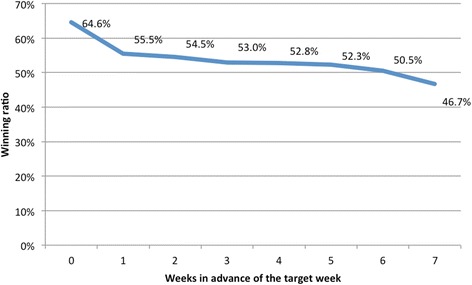
The winning ratios of EPM versus AVG for all diseases indicators and all areasᅟ

The forecasted curves of incidence of the various diseases under this study are shown from Figs. 2, 3, 4, 5 and 6. In addition, the prediction error results of two methods for five indicators of three infectious diseases are presented from Figs. 7, 8, 9, 10 and 11. If every week’s prediction on each diseases indicator was regarded as a prediction event, there were 7,945 prediction events in total. Concerning the prediction performance, for the target week (0 week in advance), EPM was more accurate in 701 out of 1,085 prediction events than AVG and the winning ratio of EPM versus AVG was 64.6 %. EPM’s winning ratio was 55.5 % for 1 week in advance, 54.4 % for 2 weeks in advance, 53.0 % for 3 weeks in advance, 52.8 % for 4 weeks in advance, 52.3 % for 5 weeks in advance and 50.5 % for 6 weeks in advance. The winning ratio of EPM was only inferior to that of AVG for the 7 weeks in advance (See Fig. 12).

### Prediction errors and winning ratios of two methods for each disease and all areas

For the prediction error of all five diseases indicators, EPM was more accurate than AVG except dengue fever confirmed cases. For the prediction of the confirmed cases of severe complicated influenza case, EPM’s prediction error was 11.955 cases while AVG’s prediction error was 18.206 cases. For the prediction of the confirmed cases of dengue fever, EPM’s prediction error was 18.131 cases while AVG’s prediction error was 16.509 cases. For the prediction of the confirmed cases of severe complicated enterovirus infection, EPM’s prediction error was 1.172 cases while AVG’s prediction error was 2.935 cases. For the prediction of the rate of enterovirus infection, EPM’s prediction error was 0.00184 % while AVG’s prediction error was 0.00411 %. For the prediction of the rate of influenza-like illness, EPM’s prediction error was 0.0104 % while AVG’s prediction error was 0.0132 % (See Table 4).

**Table 4:** Prediction error of EPM and AVG for five diseases indicators

Indicator	EPM	AVG
Confirmed cases of severe complicated influenza case	11.955	18.206
Confirmed cases of dengue fever	18.131	16.509
Confirmed cases of severe complicated enterovirus infection	1.172	2.935
Rate of enterovirus infection	0.00184	0.00411
Rate of influenza-like illness	0.0104	0.0132

### Cross analysis of prediction of five diseases and eight weeks

**Fig. 13:**
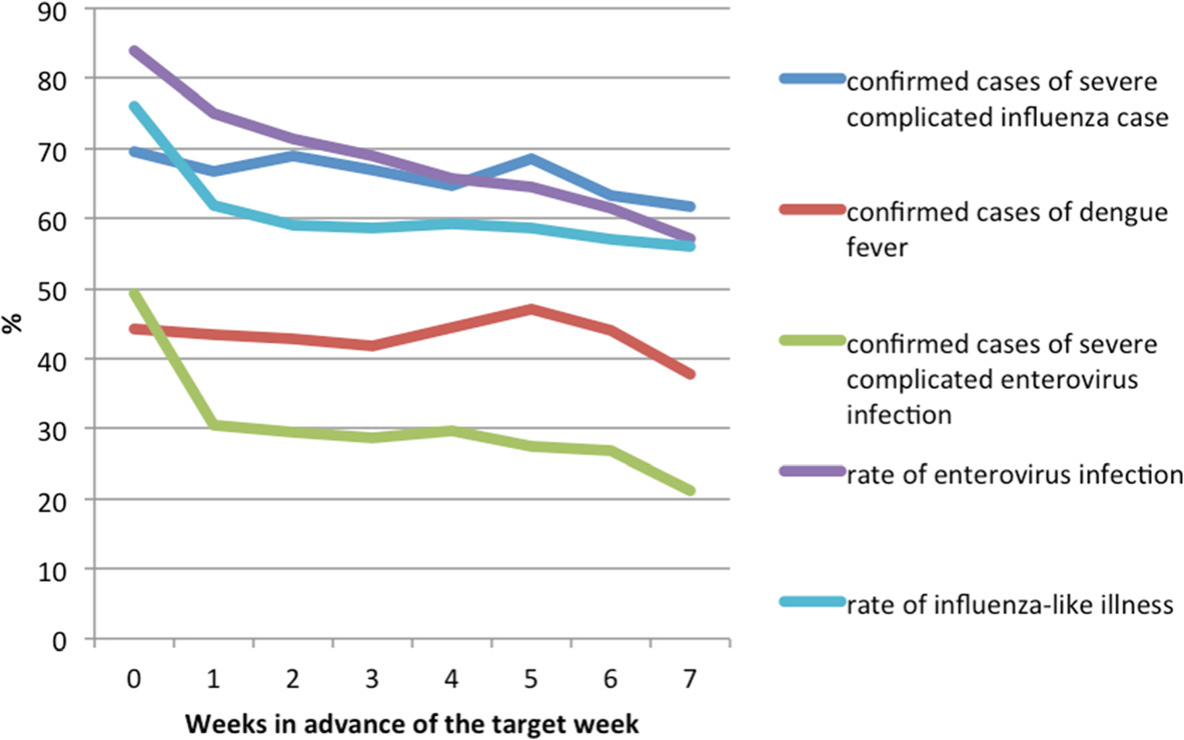
The winning ratios of EPM versus AVG for each diseases indicators and all ar﻿easᅟ

For each disease indicator, there were 1,589 prediction events, including 217 prediction events for both target week and 1 week in advance of the target week, 210 for 2 weeks in advance, 203 for 3 weeks in advance, 196 for 4 weeks in advance, 189 for 5 weeks in advance, 182 for 6 weeks in advance, 175 for 7 weeks in advance. For the prediction for the confirmed cases of severe complicated influenza case, the rate of enterovirus infection, and the rate of influenza-like illness, the winning ratios of EPM versus AVG were obviously over 50 % in all weeks. The winning ratios of EPM for these three indicators in the target week were 69.6, 83.9 and 76.0 %, respectively. Instead, for the prediction of the confirmed cases of dengue fever and the confirmed cases of severe complicated enterovirus infection, the winning ratios of EPM versus AVG were all below 50 % (See Fig. 13).

### Cross analysis of prediction of five diseases and seven areas

Table 5 shows the winning ratios of EPM versus AVG for the prediction of five diseases indicators in seven areas. First, except for the southern area and the Kaohsiung-Pingtung area (southern Taiwan), the winning ratios of EPM versus AVG for the prediction of the confirmed cases of dengue fever and severe complicated enterovirus infection were mostly less than 50 %. Second, in the southern area, the Kaohsiung-Pingtung area, and the eastern area, the winning ratios of EPM for the prediction of the rate of influenza-like illness were less than 50 %. Third, except confirmed cases of dengue fever, the highest winning ratios of EPM for predicting each indicators all occurred in the prediction of the nationwide area.

**Table 5:** The winning ratios of EPM versus AVG for five diseases indicators in seven areas

	Taipei area		Northern area		Central area		Southern area		Kaohsiung-Pingtung area		Eastern area		Nationwide area	
	All events	Target week	All events	Target week	All events	Target week	All events	Target week	All events	Target week	All events	Target week	All events	Target week
Indicator 1	71.4 %	67.7 %	65.2 %	71.0 %	56.8 %	54.8 %	60.4 %	67.7 %	62.6 %	67.7 %	63.4 %	67.7 %	81.5 %	90.3 %
Indicator 2	40.5 %	35.5 %	61.7 %	64.5 %	38.8 %	45.2 %	54.2 %	54.8 %	41.9 %	54.8 %	26.0 %	22.6 %	39.6 %	32.3 %
Indicator 3	24.2 %	48.4 %	25.1 %	35.5 %	40.1 %	54.8 %	36.1 %	54.8 %	22.0 %	64.5 %	9.3 %	12.9 %	58.6 %	74.2 %
Indicator 4	89.4 %	93.5 %	72.7 %	96.8 %	71.4 %	90.3 %	68.3 %	71.0 %	52.9 %	74.2 %	50.7 %	67.7 %	78.4 %	93.5 %
Indicator 5	67.4 %	80.6 %	71.8 %	80.6 %	68.7 %	74.2 %	40.1 %	58.1 %	44.9 %	71.0 %	61.7 %	87.1 %	73.1 %	80.6 %
Average	58.6 %	65.2 %	59.3 %	69.7 %	55.2 %	63.9 %	51.8 %	61.3 %	44.8 %	66.5 %	42.2 %	51.6 %	66.3 %	74.2 %

Note: Indicator 1: confirmed cases of severe complicated influenza case

Indicator 2: confirmed cases of dengue fever

Indicator 3: confirmed cases of severe complicated enterovirus infection

Indicator 4: rate of enterovirus infection

Indicator 5: rate of influenza-like illness

### Cross analysis of prediction of seven areas and eight weeks

Table 6 shows the winning ratios of EPM versus AVG for the prediction of all diseases indicators for eight weeks in seven areas. First, except the prediction for the target week, the winning ratios of EPM for the Kaohsiung-Pingtung and Eastern areas were less than 50 %. Second, after 4 weeks in advance of the target week in the southern area, the winning ratios of EPM were barely above 50 %. Third, the highest winning ratios of EPM for the prediction of each week all occurred in the nationwide area.

**Table 6:** The winning ratio of EPM versus AVG for the prediction of all diseases indicators for eight weeks in seven areas

	Taipei area	Northern area	Central area	Southern area	Kaohsiung-Pingtung area	Eastern area	Nationwide area
Target week	65.2 %	69.7 %	63.9 %	61.3 %	66.5 %	51.6 %	74.2 %
1 week in advance	58.7 %	60.0 %	61.3 %	54.8 %	42.6 %	43.2 %	67.7 %
2 weeks in advance	57.3 %	58.7 %	56.7 %	52.0 %	44.0 %	43.3 %	68.7 %
3 weeks in advance	58.6 %	59.3 %	53.1 %	50.3 %	40.7 %	42.1 %	66.9 %
4 weeks in advance	59.3 %	58.6 %	52.1 %	50.7 %	40.7 %	41.4 %	66.4 %
5 weeks in advance	62.2 %	58.5 %	53.3 %	48.1 %	40.7 %	37.8 %	65.2 %
6 weeks in advance	56.2 %	55.4 %	51.5 %	49.2 %	38.5 %	40.0 %	63.1 %
7 weeks in advance	49.6 %	52.0 %	46.4 %	45.6 %	42.4 %	36.0 %	55.2 %
Average	58.6 %	59.3 %	55.2 %	51.8 %	44.8 %	42.2 %	66.3 %

## Discussion

One should note that the winning ratios of EPM for the diseases have some variance in the target week. They are: (1) 69.6 % for the confirmed cases of severe complicated influenza case; (2) 44.2 % for the confirmed cases of dengue fever; (3) 49.3 % for the confirmed cases of severe complicated enterovirus infection; (4) 83.9 % for the rate of enterovirus infection; (5) 76.0 % for the rate of influenza-like illness. Among these diseases, (1), (2), and (3) are lower because they are “lagging indicators”—the data must undergo laboratorial tests to be confirmed for around one month. As this takes time, such information is hard to collect and confirmed in advance (according to Taiwan CDC).

Our EPM also improved the prediction market developed by IIM in several ways. While IIM made its prediction for one disease taking place in one State for 14 weeks, our EPM covered five diseases in seven areas in Taiwan for 31 weeks; IIM’s forecasting target was the surveillance signals, ours was the number of cases and rates. Our cases were more complicated, which increased the difficulty of prediction, yet we managed to achieve a high winning ratio when compared with the historical average.

Furthermore, the success of EPM confirms the marginal trader theory: unlike the general traders who passively take the market price, marginal traders are those who submit their orders when the market price is about to be made; they may be few in number but exert a strong impact on the eventual price [25, 26]. In EPM, 126 participants predicted 7,945 events, proving EPM to be a successful mechanism of information collection. Particularly, a few medical professionals with significant confidence of disease information were sufficient to lead the price. In fact, three top traders comprised 86.3 % of total won credits after predicting 7,945 events.

These accomplishments aside, some issues of epidemic prediction markets are worth studying in the future. First, some may question the validity of our findings by raising the possibility of self-defeating predictions—if government and people are convinced of the prediction results, would they not take preventive measures so that the original prediction becomes inaccurate? Self-defeating prediction, even if possible, is least likely to discredit prediction markets because they are more likely than any other forecasting tools to produce real-time and continuous revisions of its predictions. That is, efforts of preventive measures should be reflected in the market price.

The second issue is also about the social responses to EPM. To make their predictions more reliable, prediction markets should have as many participants as possible even if some of them are not professionals. Yet we cannot exclude the possibility that results of an open EPM can cause a dreadful response from the public if the targets are infectious diseases. How to improve public welfare without precipitating social panic is an important topic for the proponents of prediction markets to consider.

## Conclusions

EPM outperformed current methods in two ways. First, while EPM offered predictions, web search or sentinel physicians system provided only surveillance. Second, EPM predicted infectious diseases more accurately than historical averages. When compared with the historical average of the previous five years, the winning ratio of EPM on influenza, Dengue fever and enterovirus of the target weeks of 2010 is 64.6 %. There is also evidence indicating that the effectiveness of EPM improved as the forecast time drew nearer to the deadline.

In addition, the variance of EPM predictions across regions is insignificant. Across diseases, the winning ratios of the national forecasting are significantly higher than the regional ones. This is an important finding because it shows that even if participants are making imperfect predictions, they can be aggregated into more accurate ones by the mechanism of prediction markets.
